# Genetic 3′UTR variations and clinical factors significantly contribute to survival prediction and clinical response in breast cancer patients

**DOI:** 10.1038/s41598-020-62662-z

**Published:** 2020-03-31

**Authors:** Jolanta Pamuła-Piłat, Karolina Tęcza, Magdalena Kalinowska-Herok, Ewa Grzybowska

**Affiliations:** 1 Department of Genetic and Molecular Diagnostics of Cancer, Center for Translational Research and Molecular Biology of Cancer, Maria Sklodowska-Curie National Research Institute of Oncology, Gliwice, Poland; 2 Center for Translational Research and Molecular Biology of Cancer, Maria Sklodowska-Curie National Research Institute of Oncology, Gliwice, Poland

**Keywords:** Cancer genetics, Genetic interaction

## Abstract

The study describes a relationship between the 3′UTR variants, clinicopathological parameters and response to chemotherapy. We analyzed 33 germline polymorphisms in 3′UTRs of ADME genes in 305 breast cancer women treated with FAC regime. Clinical endpoints of this study were: overall survival (OS), progression-free survival (PFS), recurrence-free survival (RFS) and overall response defined as treatment failure-free survival (TFFS). The shortened OS was connected with the presence of *NR1/2* rs3732359 AA, *SLC22A16* rs7756222 CC, as well as *SLC22A16* rs9487402 allele G and clinical factors belonging to TNM classification: tumor size >1 cm, nodal involvement and presence of metastases. PFS was related to two polymorphisms *PGR* rs1824125 GG, *PGR* rs12224560 CC and *SLC22A16* rs7756222 CC as well as preexisting metastases. The RFS was shortened due to the *DPYD* rs291593 CC, *AKR1C3* rs3209896 AG and negative expression of PGR. The presence of *ALDH5A1* rs1054899 allele A, lack of pre-chemotherapy surgery and negative status of PGR correlated with worse treatment response. The germline variants commonly present in the population are important factors determining the response to treatment. We observed the effect of the accumulation of genetic and clinical factors on poor survival prognosis and overall treatment response.

## Introduction

Breast cancer usually affects women in postmenopausal period, although it is diagnosed increasingly in younger patients often in advanced clinical stages^1^. The 5-year survival is around 80% in developed countries^2^.

Therapeutic strategies for breast cancer include targeted therapy, hormone therapy, radiotherapy, surgery and chemotherapy^1,3^. Toxicity and resistance to chemotherapeutic drugs are common problems observed in patients during and after treatment. Lack of sensitivity to chemotherapy is most often associated with dysfunction of protein transporters that remove chemotherapeutic agents from the cells or DNA repair processes^4^. The response to systemic treatment is different in patients with the same type of cancer, similar stage of disease and treated with the same medications. Lack of chemotherapy response is observed as cancer-related mortality, disease progression or recurrence^5,6^.

Metabolism, absorption, distribution and excretion (ADME processes) of drugs are regulated by genes and proteins responsible for biotransformation and metabolic elimination of drugs (enzymes cytochrome P450). Also, proteins belonging to ATP binding cassette (ABC) and solute carrier (SLC) control influx or efflux of toxin and medicines and their cellular distribution^7,8^. The expression of ADME genes is regulated at the transcriptional and translational levels. In addition, they undergo posttranslational modifications. ADME dysfunction may lead to reduced drug efficacy or to an adverse drug reaction in pharmacotherapy^9,10^. Genetic and epigenetic transformations of ADME genes can modulate the activity and therapeutic effects of chemotherapy and underline interpatient differences in response to therapy^5,11–13^.

An important role in controlling gene expression is played by untranslated gene regions (UTR). 3′ untranslated regions (3′UTRs) can modify the gene expression by controlling of mRNA nuclear export, cytoplasmic localization and stability or by affecting the translational efficiency. These fragments of gene are targeted by microRNA as well as regulatory molecules^14,15^.

Polymorphisms localization in translated or regulatory sequences may influence the gene’s phenotypic effect^13,16^ and can affect proper functioning of multidrug resistance proteins^17^. However, about 93% of functional single nucleotide polymorphisms (SNPs) in the *genome-wide association study* (GWAS) catalog are in non-coding regulatory sequences of genes^17,18^, therefore they are called regulatory SNPs (rSNPs). rSNPs can influence transcription regulation or post-transcriptional expression of genes by alternative splicing or binding of transcription factors^17,19^. SNPs altering miRNA target sites in 3′UTR may create or abolish microRNA target and consequently lead to different genes activity and possible contributions to interindividual variability, observed in terms of drug response in breast cancer^7,20–22^.

The personalization of diagnostics and treatment of neoplastic diseases, including breast cancer, is gaining importance^23^. Recent research proves the clinical utility of identifying polymorphisms for effective stratification of risk and treatment of breast cancer^23,24^. Genotype analysis should be useful in prediction the therapeutic decision. However, little is known about how information about the genetic profile of patients is interpreted in connection with clinical decision-making^25^.

In this study we performed genetic investigation of 33 germline polymorphisms within 3′UTRs of ADME genes in breast cancer patients treated with FAC regime (doxorubicin, 5′-fluorouracil and cyclophosphamide) in relation to overall survival (OS), progression-free survival (PFS), recurrence-free survival (RFS) and overall response to treatment.

## Materials and Methods

### Patients

305 breast cancer women were included in this study. All individuals were Caucasians from Silesian Voivodeship (southern Poland). The chosen group was composed only of non-carriers of germline mutations c.5266dupC, c.181 T > G, c.68_69delAG, c.4035delA in *BRCA1* and c.9403delC, c.5946delT in *BRCA2* gene. Patients who were qualified for the study were diagnosed from 1997 to 2012. The observation ended on 30th August 2017. Detailed information about the study group is provided in Table 1.

**Table 1:** Characteristics of breast cancer patients group.

	Characteristics	n (%)
**General**	**Age at diagnosis** **(years)**	
	• ≤39	26 (8.0)
	• 40–50	222 (68.5)
	• ≥61	76 (23.5)
	Mean age at diagnosis in years (min-max)	54.7 (22.4–79.0)
	**Year of diagnosis**	
	• 1997–2004	15 (4.6)
	• 2005–2009	289 (89.2)
	• 2010–2012	20 (6.2)
	**Histopathology**	
	• invasive ductal carcinoma	230 (71.1)
	• invasive lobular carcinoma	22 (6.8)
	• carcinoma mixed type	6 (1.8)
	• other	29 (8.9)
	• unspecified	37 (11.4)
	**Tumor grade**	
	• G1	39 (12.0)
	• G2	71 (21.9)
	• G3	83 (25.8)
	• Bloom I	5 (1.5)
	• Bloom II	12 (3.7)
	• Bloom III	12 (3.7)
	• Unspecified	102 (31.4)
**Receptors**	**Estrogen receptor status**	
	• Negative	115 (35.5)
	• Positive	190 (58.6)
	• Unspecified	19 (5.9)
	**Progesterone receptor status**	
	• Negative	133 (41.0)
	• Positive	172 (53.1)
	• Unspecified	19 (5.9)
	**HER2 status**	
	• Negative	103 (31.8)
	• Positive	167 (61.5)
	• Unspecified	54 (16.7)
	**triple-negative breast cancer (TNBC)**	37 (11.4)
**TNM staging**	**Tumor (T)**	
	• 0	2 (0.6)
	• 1	43 (13.3)
	• 2	97 (29.9)
	• 3	52 (16.0)
	• 4	82 (25.3)
	• Unspecified	50 (15.5)
	**Nodes (N)**	
	• 0	85 (26.2)
	• 1	108 (33.3)
	• 2	67 (20.7)
	• 3	19 (5.8)
	• 4	1 (0.3)
	• Inspecified	44 (13.6)
	**Metastases (M)**	
	• 0	258 (79.6)
	• 1	25 (7.7)
	• Unspecified	42 (12.7)
	**Metastases locations**	
	• Liver	8 (32.0)
	• Lungs	3 (12.0)
	• bones and lungs	3 (12.0)
	• bones	2 (8.0)
	• other	9 (36.0)
**Therapy**	**Surgery**	
	• amputation	187 (57.7)
	• conserving surgery. including:	87 (26.8)
	• with radicalization	14 (4.3)
	• without radicalization	73 (22.5)
	• none	50 (15.5)
	**Hormonotherapy**	
	• yes	204 (63.0)
	• no	120 (37.0)
	**Immunotherapy (Herceptine)**	
	• yes	36 (11.1)
	• no	288 (88.9)
	**Chemotherapy FAC**	
	• adjuvant	136 (42.0)
	• neoadjuvant	188 (58.0)
	mean numer of cycles (range)	6.1 (3–9)
	**Radiotherapy**	
	• yes	265 (81.8)
	• no	59 (18.2)
	• brachytherapy	7 (2.2)
	mean radiation dose in Gy (range)	50.2 (20–70)
	mean radiation dose in brachytherapy (range)	14 (10–30)
**FOLLOW-UP**	**Deaths**	
	• yes	98 (32.2)
	• no	207 (67.8)
	median OS in months (min-max)	87.0 (4.3–177.7)
	**Progression**	
	• yes	107 (35.1)
	• no	198 (64.9)
	median PFS in months (min-max)	78.0 (0.9–176.7)
	**Progression- locations of metastases**	
	• bones	29 (27.1)
	• multiorgan spread	33 (30.9)
	• lungs	9 (8.4)
	• liver	8 (7.5)
	• lymph nodes	9 (8.4)
	• tumor growth	10 (9.3)
	• central nervous system	5 (4.7)
	• skin	3 (2.8)
	• eye socket	1 (0.9)
	**Recurrence**	
	• yes	14 (4.6)
	• no	291 (95.4)
	median RFS in months (min-max)	82.8 (0.5–176.7)
	**Metachronous primary breast cancer**	
	• yes	11 (3.6)
	• no	294 (96.4)
	median survival to next breast cancer diagnosis in months (min-max)	82.6 (0.5–176.7)
**Baseline Blood Tests Results**	white blood cells (10^3^/µl)	6.90 ± 1.92
	neutrophiles (10^3^/µl)	4.04 ± 1.58
	thrombocytes (10^3^/µl)	273.5 ± 74.10
	monocytes (10^3^/µl)	0.690 ± 2.30 H
	reticulocytes (10^3^/µl)	64.94 ± 20.67
	RDW (%)	14.59 ± 10.03 H
	hemoglobin (g/dl)	13.80 ± 1.28
	creatinine (µmol/l)	71.98 ± 14.00
	bilirubin. total (µmol/l)	10.76 ± 12.16
	ALAT (U/l)	21.13 ± 13.52
	AspAT (U/l)	21.07 ± 12.42
	ALP (U/l)	76.7 ± 40.78

HER2- human epidermal growth factor receptor-2; OS- overall survival; PFS- progression-free survival; RFS- recurrence-free survival; RDW- Red (cell) Distribution Width; ALAT- alanine transaminase; AspAT- aspartate transaminase; ALP- alkaline phosphatase; H- high.

### Sample collection and DNA extraction

Peripheral blood (10 ml) from each patient was collected and stored (−80 °C) in EDTA- containing tubes. Genomic DNA used for genotyping studies was extracted using commercial DNA isolation kits. DNA samples were stored at −20 °C.

### SNP selection and genotyping

We selected 33 SNPs according to the following criteria: (1) the minor allele frequencies (MAF) of these SNPs were ≥0.05 in the Caucasian population and: (2) the selected SNPs were located in 3′UTRs of ADME genes^26^. In our study, we focused on SNPs in regulatory regions of genes encoding proteins involved in pharmacokinetics of 5-fluorouracil, doxorubicin, cyclophosphamide (FAC regime). We chose genes encoding proteins which participate in drug transport (*ABCA1, ABCC4, ABCC1, ABCB1, ABCC5, SLC22A16, RALBP1*), metabolism (*AKR1C3, ALDH5A1, CBR1,CYP1A2, CYP2E1, CYP1B1, NOS3, SULT4A1, UGT2B15, UGT2B4, DPYD, GSTM3*), drug-induced damage repair (*ERCC1, ERCC4*) and nuclear receptors (*NR1/2, PGR*). Selection of SNPs was based on literature data as well as on online database search: dbSNP^27^, PubMed^28^ and Ensembl BioMart^29^. SNPs id and their frequencies were obtained from International 1000 Genomes Project^30^ or Ensembl BioMart^29^.

A PCR–based restriction fragment-length polymorphism analysis was performed to determine the genotypes for all studied SNPs. The genotyping methods for polymorphisms in all genes studied were developed for this study. Primers were designed with Primer 3^31^ and Primer-BLAST^32,33^ open-access web-based tools. Polymerase chain reactions (PCR) were carried out in a final volume of 15 µl using Perpetual Taq Polymerase reagents (EURx, Poland) and Eppendorf thermal cyclers (Eppendorf, Germany). Temperature conditions of the PCR reactions and composition of the reaction mixtures are available on request. Restriction fragments length polymorphism (RFLP) methods were designed using the WatCut^34^ or NEBCutter v2.0^35^ web-based tools. Detection of SNPs was carried out with restriction enzymes in accordance with the manufacturer’s instructions (EURx, Poland; New England BioLabs, USA). Electrophoresis of PCR products and digestion products was performed on agarose gel stained with ethidium bromide. Genotype detection was confirmed on selected samples by Sanger sequencing (Genomed, Poland). The details of the genetic variants studies and primer sequences and restriction enzymes present Table 2.

**Table 2:** Details of the genetic variants analyses, primer sequences and restriction enzymes used.

Function	Gene	SNP ID	Alleles wt/v	Primer sequences 5′→3′	Restriction enzyme
**Transporters**	*ABCA1*	rs4149339	C/T	F: TCA GTC ATG ACT AGT GCC TA R: ATC CAA TAT CTG CAA AGC CA	SspI
	*ABCC4*	rs9516521	C/T	F: AACCAAAAGGCTTACAGTCA R: CCTGAAGGCTTCTTGTTAGA	HaeIII
	*ABCC1*	rs212090	A/T	F: TCT ACC AGT TCT CGT TTT GG R: AAG CTT CAT AAC CAT GAG CA	MboI
		rs212091	A/G	F: GATTCCACTTTGGGCTCTAA R: TTTTAAGTACTGTTCCGGGG	DdeI
		rs129081	C/G	F: GCAAGTCTTTGAGATGCTTC R: TTGCTCACTCTCAGTCTCTA	MlyI
		rs3743527	C/T	F: GACTTCTGGAGGAATTGGTT R: AGTTCCAGGTCCAGGTTAG	BseRI
	*ABCB1*	rs17064	A/T	F: TTTTCAATGGTCAGTGTCCA R: GGTTCTGTAAGACCCAATGT	ApoI
	*ABCC5*	rs3805114	A/C	F: TTAGCATGTTTGCTGAACAC R: ATTTTCAATGGGTGAATCTG	MnlI
	*SLC22A16*	rs7756222	C/T	F: AGG GTT TCT GGA AGC TTT AG R: TGC CCC ATA AAG TAG CAA TT	HhaI
		rs9487402	T/G	F: AGG GTT TCT GGA AGC TTT AG R: TGC CCC ATA AAG TAG CAA TT	AluI
	*RALBP1*	rs12680	C/G	F: GGA GAT TGA GTA CTC TGC AG R: TCC CCA ATG CTA AAT GTA CC	AvaII
**Drugs metabolizers**	*AKR1C3*	rs3209896	A/G	F: CTC CCC TAG TAA TGG AGT CA R: CAG GCA AAT CAC ACA GTT TT	MnlI
	*ALDH5A1*	rs1054899	A/C	F: TCA AGT ATG TGT GTT ACG GG R: ACG GAC AAT TCT GTG CTA TT	MboI
	*CBR1*	rs20572	C/T	F: GTG GTG AAC GTA TCT AGC AT R: TGC ATC AGA GGA AAT CAC AA	BfaI
	*CYP1A2*	rs17861162	C/G	F: CCATGTTGGCTAGACTAGTC R: GGGTCAAACAACATGAAAGG	BglI
	*CYP2E1*	rs2480256	C/T	F: GCACTCCATCCTGGTCAACA R: ACGAGTGTGTGCTGGAGAAG	TagI
	*CYP1B1*	rs162562	A/C	F: CTGCTTCTCAATTAGCGTTT R: GAAAAGGGAATTTCTGGTCT	MboII
	*NOS3*	rs2566508	G/T	F: CTA GAC TCA AGC AAT CCT CC R: GGC TGT AGG TTA TAT GGG TG	DdeI
	*SULT4A1*	rs138057	A/G	F: CAGACTTCCCTCAGCACAT R: AGTGGTGAGAACAGAGGATG	MnlI
	*UGT2B15*	rs3100	T/C	F: GATCCAGTACCACTCTTTGG R: GCATCCAGTAACTCGTCATT	MmeI
	*UGT2B4*	rs1131878	A/G	F: ATTGTTCCAATTCACAGGTT R: AACCAAAAACCAGTTGTCAC	AluI
	*DPYD*	rs291592	A/G	F: TGT GAC AGT TTC CAA ATT GC R: CAA ACT GAT TTG GCA CAC TT	HpyCH4III
		rs291593	C/T	F: TGT GAC AGT TTC CAA ATT GC R: CAA ACT GAT TTG GCA CAC TT	NdeI
	*GSTM3*	rs3814309	C/T	F: GAGACCTTCCCTCTCTATGT R: CTCATGCATTGCTTGTGTAG	RsaI
**Dna Repair**	*ERCC1*	rs1046282	A/G	F: CAGGATTAATGGTCGTGGAT R: GTTAAGACCAATGGACCGAT	HaeIII
		rs3212986	G/T	F: CAG CTC CTT TAA TGA CTG GT R: AAG AAG CAG AGT CAG GAA AG	MboII
	*ERCC4*	rs2276464	G/C	F: TTGTCTACAAAGGAGCCTTC R: CTGGACTTAGGGATCTCTGA	SpeI
		rs2276466	C/G	F: TTCATGGAGCCTTCCTACTA R: CACAGGATGGTGAGAAATGA	MnlI
		rs4781563	A/G	F: ATCATGGTGCCTCTTTTGAT R: CCAAATGAGTTTTCTGAGCC	DdeI/HaeIII
**Nuclear receptors**	*NR1/2*	rs3732359	A/G	F: CAGTCTGTAGGGAGTGAAGC R: GATGCAGAGACACAGAATGA	FokI
	*PGR*	rs1824125	G/T	F: AACTTGGCGCTTAATAATCT R: AGGTGAGATTCAGACAAGTA	AvaII
		rs561610	C/T	F: TCC AAA ACC CTT CTG GAA AA R: AGA CTG AAG AAG AAA GCC AG	MboI
		rs11224560	C/T	F: CTG GCT TTC TTC TTC AGT CT R: TAT TGG AGC ACC TAA GAG GA	NlaIII

The genotyping methods for polymorphisms in all genes were developed for this study; SNP-single nucleotide polymorphism; wt-wild type; v-SNP variation.

### Statistical analyses

For the rs17064 in *ABCB1* gene, rs12680 in *RALBP1*, rs17861162 in *CYP1A2* gene, rs3805114 in *ABCC5*, rs20572 in *CBR1* we found only cases of frequent homozygote and heterozygote. The carriers of rare homozygotes were not found in examined set of patients. Statistical calculations for these genes were done between reference homozygotes and heterozygotes.

The main purpose of the study was describing the relationship between 3′UTR SNPs, clinical parameters and response to chemotherapy. Survival prediction and clinical response were defined and calculated as follows: (a) Overall Survival (OS) - fine-needle aspiration (FNA) confirmed breast cancer diagnosis to date of death or to date of the last follow-up; (b) Progression-Free Survival (PFS) - initiation of chemotherapy to progression or to date the last follow-up; (c) Recurrence-Free Survival (RFS) - start of chemotherapy to date of recurrence or to date of the last follow-up; recurrence or progression were affirmed by the magnetic resonance imaging (MRI), computed tomography (CT) or ultrasound examination; (d) metachronous breast cancer - from time point of beginning of chemotherapy to the date of FNA of the second tumor or to the to date of the last follow-up evaluation; (e) treatment failure-free survival (TFFS) - start of chemotherapy to disease progression, death, recurrence or metachronous breast cancer, that occurred during 10 months from the initiation of FAC regime, or to the date of recent medical check-up.

Hardy-Weinberg Equilibrium (HWE) with the χ^2^ test was used to establish the genotype frequencies. Pearson χ^2^ and Fisher two-way exact tests were applied to establish relationship between 3′UTR variants, clinical-pathological parameters and response to chemotherapy. All genetic and clinical-pathological factors that correlated with treatment response, death, progression, recurrence and metachronous primary breast cancer with p ≤ 0.10 calculated in univariate analysis was interpreted as trend. Risk factors with p ≤ 0.10 have a possible but poor effect on the outcome of the treatment. Univariate analysis results were used as starting point for multivariate calculations. Stepwise regression made possible to obtain data on independent prognostic factors (p ≤ 0.05) of FAC chemotherapy. Cumulative analyzes enabled us to estimate the risk of non-response to the therapy for two or more independent factors, as described previously^36^.

Survival analysis (OS, PFS, RFS, TFFS) were evaluated by the Kaplan-Meier method, with the log-rank test for comparisons of subgroups. P ≤  0.05 was considered as significant. Relative risk of OS, PFS, RFS, TFFS was calculated and expressed as hazard ratios (HRs), 95% confidence intervals (95%CIs) and p-value. The data were analyzed using Statistica v10.0 software (StatSoft).

### Ethical approval and consent to participate

Each participant completed a standard informed consent form and agreed to have their samples used for research purposes. The study was approved by local Bioethical Commission at Maria Sklodowska-Curie National Research Institute of Oncology, Gliwice, Poland. Approval No. KB/430-68/12.

### Consent for publication

All authors read and approved the final manuscript.

## Results

### Association of SNPs and clinical factors with survival

For each survival analysis- OS, PFS and RFS, there were several factors, both genetic and clinical, influencing the risk of death, progression and recurrence, respectively (Table 3).

**Table 3:** Univariate analyses of associations between genetic variants, clinical characteristics and survival.

Analysis	Variable	Event*		Event* risk HR (±95% CI)	P
		absent n (%)	present n (%)		
Overall Survival (OS)	*NR1/2* rs3732359				
	AG/GG	120 (59.1)	39 (40.2)	1 (ref.)	
	AA	83 (40.9)	58 (59.8)	1.82 (1.24–2.80)	0.003
	*SLC22A16* rs7756222				
	CT/TT	144 (69.9)	57 (58.2)	1 (ref.)	
	CC	62 (30.1)	41 (41.8)	1.58 (1.05–2.36)	0.027
	*SLC22A16* rs9487402				
	TT	112 (54.6)	39 (39.8)	1 (ref.)	
	TG/GG	93 (45.4)	59 (60.2)	1.72 (1.14–2.59)	0.009
	T [tumor; TNM]				
	1	37 (20.6)	6 (7.0)	1 (ref.)	
	>1	143 (79.4)	80 (93.0)	3.18 (1.38–7.32)	0.006
	N [nodes; TNM]				
	0	61 (33.7)	19 (22.1)	1 (ref.)	
	>0	120 (66.3)	67 (77.9)	1.80 (1.06–3.03)	0,027
	M [metastases; TNM]				
	No	178 (99.4)	67 (76.1)	1 (ref.)	
	Yes	1 (0.6)	21 (23.9)	9.74 (5.74–16.54)	<0.00001
Progression-Free Survival (PFS)	*SLC22A16* rs7756222				
	CT/TT	139 (70.2)	62 (58.5)	1 (ref.)	
	CC	59 (29.8)	44 (41.5)	1.57 (1.07–2.32)	0.021
	*PGR* rs1824125				
	GT/TT	54 (27.7)	18 (17.1)	1 (ref.)	
	GG	141 (72.3)	87 (82.9)	1.76 (1.06–2.95)	0.029
	*PGR* rs11224560				
	CT/TT	54 (27.7)	18 (17.1)	1 (ref.)	
	CC	141 (72.3)	87 (82.9)	1.76 (1.06–2.92)	0.029
	M [metastases; TNM]				
	No	168 (98.2)	77 (80.2)	1 (ref.)	
	Yes	3 (1.8)	19 (19.8)	11.20 (6.46–19.41)	<0,00001
recurrence-free survival (RFS)	*DPYD* rs291593				
	CT/TT	159 (54,8)	3 (21,4)	1 (ref.)	
	CC	131 (45,2)	11 (78,6)	5,89 (1,29–26,88)	0,022
	*AKR1C3* rs3209896				
	AA/GG	153 (53.1)	3 (21.4)	1 (ref.)	
	AG	135 (46.9)	11 (78.6)	5.49 (1.20–25.05)	0.028
	*PR* status				
	Positive	161 (58.5)	2 (15.4)	1 (ref.)	
	Negative	114 (41.5)	11 (84.6)	7.23 (1.56–33.51)	0.011

HR- hazard ratio; 95%CI- confidence interval; bolded numbers indicate results with p < 0.05; bolded bases indicate reference genotype; *OS: death; PFS: progression; RFS: recurrence.

The shortened overall survival and elevated risk of death was connected with the presence of *NR1/2* rs3732359 common homozygote AA (HR 1.82; 1.24–2.80; p = 0.003), common homozygote *SLC22A16* rs7756222 CC (HR 1.58; 1.05–2.36; p = 0.027), as well as with the presence of *SLC22A16* rs9487402 allele G (HR 1.72; 1.14–2.59; p = 0.009). Clinical factors belonged to tumors malignant classification (TNM): tumor size >1 cm (HR 3.18; 1.38–7.32; p = 0.006), nodal involvement (HR 1.80; 1.06–3.03; p = 0.027) and presence of metastases (HR 9.74; 5.74–16.54; p < 0.00001).

**Figure 1:**
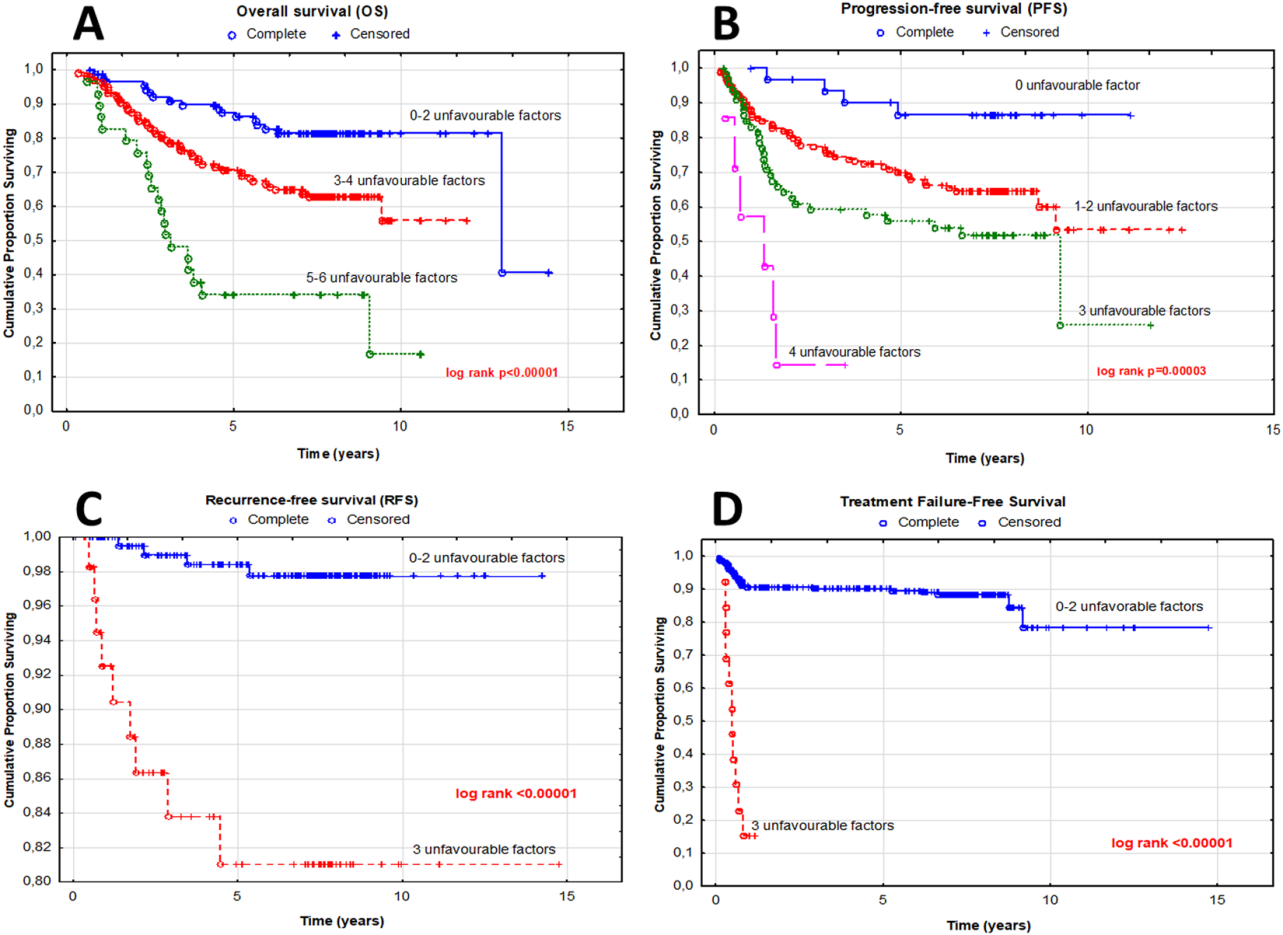
Kaplan-Meier survival curve for FAC treatment analysis of breast cancer patients. (**A**) Overall survival and cumulative risk of death for unfavorable OS factors, (**B**) progression-free survival and cumulative risk of disease progression for unfavorable PFS factors, (**C**) recurrence-free survival and cumulative risk of disease recurrence for unfavorable RFS factors. (**D**) Treatment Failure-Free Survival (TFFS) curve presents association between number of unfavorable genotypes, clinical parameters and lack of treatment response risk and survival of breast cancer patients; HR-hazard ratio.

In the cumulative analyses, the presence of growing number of high-risk factors was reflected in the increasing risk of death, from HR 4.40; 1.33–14.49; p = 0.015 for the three factors to HR 10.88; 9.01–185.47; p < 0.00001 for the carriers of all six of them. It should be noted that in the group of non-carriers there were no incidences of death, therefore the reference group in this analysis was constructed for the non-carriers and the carriers of one single factor combined. Also, in these conditions the impact of concomitant presence of two high-risk death factors was not statistically significant, placing itself in the p-value range established for the trend. For clearer image and easier interpretation, the cumulative groups underwent further fusion, based on the closeness of the HRs and p-value. In the result, three groups of patients were obtained: the carriers of 0–2 factors (reference group), 3–4 and 5–6 factors. In these groups the death risk was gradually elevated, from HR 2.36; 1.34–4.14; p = 0.003 to HR 6.34; 3.27–12.30; p < 0.00001 (Table 4, Fig. 1A).

**Table 4:** Multivariate analyses of associations between unfavorable genotypes and the treatment response risk.

Analysis	Unfavorable genotypes and clinical factors		Number of unfavorable factors	Event*		median survival (months)	log rank p	Event* risk HR (±95% CI)	P
				absent n (%)	present n (%)				
Overall Survival (OS)			0	3 (1.7)	0 (0.0)	94.33		1 (ref.)	
			1	29 (16.6)	4 (4.7)	91.77			
	*NR1/2* rs3732359	AA	2	43 (24.6)	13 (15.1)	91.31		3.02 (0.86–10.59)	0.085
	*SLC22A16* rs7756222	CC	3	61 (34.9)	27 (31.4)	86.51	**<0.00001**	**4.40** (1.33–14.49)	**0.015**
	*SLC22A16* rs9487402	TG/GG	4	29 (16.6)	22 (25.6)	72.75		**6.59** (1.97–22.02)	**0.002**
	T [tumor; TNM]	>1	5	10 (5.7)	16 (18.6)	40.39		**12.03** (3.50–41.40)	**0.00008**
	N [nodes; TNM]	>0	6	0 (0.0)	4 (4.7)	21.26		**10.88** (9.01–185.47)	**<0.00001**
	M [metastases; TNM]	yes	0–2	75 (42.9)	14 (16.9)	92.18		1 (ref.)	
			3–4	90 (51.4)	49 (59.0)	84.33	**<0.00001**	**2.36** (1.34–4.14)	**0.003**
			5–6	10 (5.7)	20 (24.1)	36.31		**6.34** (3.27–12.30)	**<0.00001**
			0	28 (16.7)	4 (4.5)	89.82		1 (ref.	
			1	14 (8.3)	9 (10.2)	79.61		**3.71** (1.14–12.06)	**0.029**
Progression-Free	*SLC22A16* rs7756222	CC	2	88 (52.4)	44 (50.0)	77.79	**0.00007**	**3.20** (1.15–8.91)	**0.026**
Survival (PFS)	*PGR* rs1824125	GG	3	37 (22.0)	31 (35.2)	51.60		**5.15** (1.82–14.60)	**0.002**
	*PGR* rs11224560	CC	4	1 (0.6)	6 (6.8)	16.03		**19.18** (5.34–68.83)	**<0.00001**
	M [metastases; TNM]	yes	0	28 (16.7)	4 (4.3)	89.82		1 (ref.)	
			1–2	102 (60.7)	53 (56.4)	78.00		**3.28** (1.19–9.06)	**0.022**
			3	37 (22.0)	31 (35.2)	51.60	**0.00003**	**5.15** (1.82–14.60)	**0.002**
			4	1 (0.6)	6 (6.8)	16.03		**19.18** (5.34–68.83)	**<0.00001**
			0	77 (28.1)	1 (7.7)	81.26		1 (ref.)	
			1	63 (23.0)	1 (7.7)	81.69		1.34 (0.08–21.38)	0.837
Recurrence-Free	*DPYD* rs291593	CC	2	83 (30.3)	2 (15.4)	88.69	**0.00004**	0.89 (0.05–14.29)	0.937
	*AKR1C3* rs3209896	AG	3	51 (18.6)	9 (69.2)	52.34		**13.14** (1.64–105.14)	**0.015**
Survival (RFS)	*PR* status	negative	0–2	223 (81.4)	4 (30.8)	83.93	**<0.00001**	1 (ref.)	
			3	51 (18.6)	9 (69.2)	52.34		**10.60** (3.26–34.44)	**0.00009**

HR- hazard ratio; 95%CI- confidence interval; bolded numbers indicate results with p < 0.05; *OS: death; PFS: progression; RFS: recurrence.

From the genetic side, the high risk of progression and reduced PFS was related to two polymorphisms on progesterone receptor gene (PGR) rs1824125 GG (HR 1.76; 1.06–2.95; p = 0.029), *PGR* rs12224560 CC (HR 1,76; 1.06–2.92; p = 0.029), as well as to common homozygote CC of the variant *SLC22A16* rs7756222 CC (HR 1.57; 1.07–2.32; p = 0.021). Preexisting metastases (HR 11.20; 6.46–19.41; p < 0.00001) constitute the strongest single clinical high-risk factor for progression, (Table 3). The cumulative analysis showed shortened progression-free survival with the growing number of unfavorable factors, from HR 3.71; 1.14–12.06; p = 0.029 for single factor to HR 19.18; 5.34–68.83; p < 0.00001 for the presence of all four. Similarly as in the OS cumulative analysis, we fused the groups with similar risk of progression to establish a more interpretation-friendly model (Table 4, Fig. 1B).

The recurrence-free survival was shortened for the *DPYD* rs291593 CC, *AKR1C3* rs3209896 AG and negative expression of progesterone receptor. Cox proportional hazard analysis revealed their strong but similar impa ct on risk of the recurrence of the disease- HR 5.89; 1.29–26.88; p = 0.022, HR 5.49; 1.20–25.05; p = 0.028 and HR 7.23; 1.56–33.51; p = 0.011, respectively (Table 3). In the cumulative analysis, the only statistical significance was obtained for the carriers of all three recurrence high-risk factors- HR 13.14; 1.64–105.14; p = 0.015. The confrontation of this group with the others combined (0–2) slightly reduced the risk but at the same time powered up the statistical significance- HR 10.60; 3.26–34.44; p = 0.00009 (Table 4, Fig. 1C).

### Effect of genetics and clinical factors on treatment response

Multivariate analysis performed for all selected genotypes revealed one genetic and two clinical independent prognostic factors for the response to FAC chemotherapy. Patients carrying the heterozygous and rare homozygous genetic variants (CA/AA) of *ALDH5A1* variant rs1054899 presented a significantly increased risk of lack of treatment response (OR 2.74; 1.25–6.02; p = 0.012) compared with reference homozygous allele (CC) group. Analysis performed for clinical parameters revealed that lack of pre-chemotherapy surgery and negative status of progesterone receptor were risk factors associated with lack of treatment response, - OR 9.95; 4.54–21.80; p < 0.00001 and OR 2,49; 1.16–5.34; p = 0.018, respectively (Table 5).

**Table 5:** Multivariate analysis of the genetic variants, clinical parameters and lack of treatment response.

Analysis	Variable	Lack of treatment response		Lack of treatment response risk OR (±95% CI)	P
		no n (%)	yes n (%)		
Response	*ALDH5A1* rs1054899				
	**CC**	137 (53.5)	15 (34.1)	1 (ref.)	
	CA/AA	119 (46.5)	29 (65.9)	**2.74** (1.25–6.02)	0.012
	*Surgery*				
	Yes	236 (90.4)	21 (47.7)	1 (ref.)	
	No	25 (9.6)	23 (52.6)	**9.95** (4.54–21.80)	<0.00001
	*PR* status				
	Positive	149 (60.6)	14 (33.3)	1 (ref.)	
	Negative	97 (39.4)	28 (66.7)	**2.49** (1.16–5.34)	0.018

OR- odds ratio; 95%CI- confidence interval; bolded numbers indicate results with p < 0.05; PR- progesterone receptor.

The cumulative analysis showed that the simultaneous presence of two of those factors was responsible for nearly 7-fold increase in lack of treatment response risk (OR 6.87; 1.90–24.81; p = 0.003), while the carriers of all three factors resulted in drastic strong risk elevation to OR 135.67 (19.80–929.78; p < 0.00001). This bad prognosis was maintained also after confrontation with other groups combined (0–2)- OR 42.40; 8.92–201.64; p < 0,00001 (Table 6).

**Table 6:** The association between accumulations of unfavorable genetic variant and clinical parameters.

	Unfavorable genotypes and clinical factors	Number of unfavorable factors	Lack of treatment response		Logistic Regression		Treatment Failure Free Survival (TFFS)			
			no n (%)	yes n (%)	Lack of treatment response risk OR (±95% CI)	P	median survival (months)	log rank p	Event* risk HR (±95% CI)	p
*ALDH5A1* rs1054899 *surgery* *PGR* status	CA/AA no negative	0	74 (30.7)	3 (7.1)	1 (ref.		82.59	**<0.00001**	1 (ref.	
		1	104 (43.2)	11 (26.2)	2.61 (0.91–9.76)	0.152	85.44		2.46 (0.69–8.84)	0.167
		2	61 (25.3)	17 (40.5)	**6.87** (1.90–24.81)	**0.003**	37.59		**6.55** (1.92–22.38)	**0.003**
		3	2 (0.8)	11 (26.2)	**135.67** (19.80–929.78)	**<0.00001**	5.67		**54.17** (14.74–199.14)	**<0.00001**
		0–2	239 (99.2)	31 (73.8)	1 (ref.)		79.44	**<0.00001**	1 (ref.)	
		3	2 (0.8)	11 (26.2)	**42.40** (8.92–201.64)	**<0.00001**	5.67		**17.08** (8.26–35.36)	**<0.00001**
		0–1	178 (73.9)	14 (33.3)	1 (ref.)		83.51	**<0.00001**	1 (ref.)	
		2–3	63 (26.1)	28 (66.7)	**5.65** (2.79–11.45)	**<0.00001**	25.84		**5.20** (2.78–9.91)	**<0.00001**

Lack of treatment response risk and survival of patients defined as the median TFFS survival expressed in months. OR- odds ratio; 95%CI- confidence interval; HR- hazard ratio; * event- death. progression. recurrence and/or secondary metachronous breast cancer.

To check for possible application of the cumulative model in predicting long-term survival of patients, we estimated treatment failure-free survival (TFFS). TFFS median reduction was associated with the increasing number of high-risk unfavorable genetic and clinical factors - from 82.59 months for non-carriers to 5.67 months for the presence of all three factors. Consequently, in the results of Cox logistic regression the group with worst prognosis had also the highest risk of event during observation - HR 54.17; 14.74–199.14; p < 0.00001 (Table 6, Fig. 1D). Such strong impact was maintained also after confrontation with groups 0–2 (HR 17.08; 8.26–35.36; p < 0.00001), as well as after fusion with the carriers of two factors (HR 5.20; 2.78–9.91; p < 0.00001) (Table 6).

## Discussion

In this study, we investigated the association between the 3′UTR SNPs of genes involved in FAC drugs’ transport, metabolism, regulation of detoxification pathways, nuclear receptors, clinical parameters and the overall response to FAC chemotherapy. The results suggest that the risk of death, disease progression or recurrence of breast cancer is modified by genetic variants of nuclear receptors (*NR1/2, PGR*), genes engaged in main metabolic pathway of doxorubicin (*SLC22A16*) and doxorubicin-progesterone-related gene (*AKR1C3*). SNPs within *DPYD* and *ALDH5A1* genes were significantly associated with the increase risk of RFS and treatment response/TFFS analyses. The clinical prognostic factors that influenced survival and treatment response in our study have grouped themselves in three categories - the components of TNM staging in OS and PFS, tumor progesterone receptor status in RFS and response/TFFs, and the implementation of surgery procedures (regardless of their extent) in treatment response/TFFS analyses.

The family of PXR plays a regulatory function in reference to enzymes of I phase (cytochrome P450 enzymes CYP3A4, CYP2B6, CYP2C9, and CYP2C19), phase II enzymes (UGT1A1, UGT1A2, SULT2A) and phase III transporters (ABCB1, OATs, MRP3)^37–42^. 3′UTR of pregnane X receptor (*NR1/2/*PXR2), a key component of xenobiotic sensor, are targets for the presence of several microRNAs, including miR-362-5p, miR-500b-5p and miR-501-5p, which suggests the importance of epigenetic regulation of *NR1/2* expression^21^. Our observations of the correlations between the said gene’s 3′UTR genetic variant and patients survival seem to support such statement, however the exact genetic-clinical linkage is yet to be confirmed. Reuter and colleagues searched for such correlation, and while they showed an impact of NR1/2 polymorphisms on protein expression in blood and tissue samples of head and neck squamous cell carcinoma (HNSCC) patients, they were unable to demonstrate their influence on overall survival times^43^.

Regardless of the inconsistency of research results it is plausible that genetic variations within *NR1/2* that influence protein expression or activity, have significant clinical effects of diverse character^38^. SNPs within coding sequence of *NR1/2* gene have correlated with risk of overall cancer^44^, progression of AIDS^45^, were the potential risk factor of drug resistance in epilepsy^46^ as well as of hematological toxicity induced by irinotecan in colorectal cancer patients^47^. Genetic variants in the 3′UTR region of *NR1/2* affect transport, localization and the stability of NR1/2 mRNA^14,40^. Several studies have confirmed the influence of the 3′UTR SNPs of *NR1/2* on the treatment effects with a resultant increase in resistance to chemotherapy also in breast cancer patients^39–42^. The group of Oleson associated rs3732359 and rs3732360 of *NR1/2* with higher CYP3A activity *in vivo*. The CYP3A4 is the major drug metabolizing enzyme and downstream effector gene of NR1/2. Furthermore, Oleson *et al*. found that variants rs3732359 and rs3732360 of *NR1/2* exhibited higher median oral midazolam clearance compared with homozygous reference genotypes for these SNPs^38^. In our group of patients, the presence of rs3732359 AA *NR1/2* was an independent predictor of OS. In univariate analyses carriers with genotype AA present a nearly 2-fold increase in the risk of death compared to patients *NR1/2* rs3732359 AG/GG. That observation suggested association of rs3732359 AA *NR1/2* with worse survival prognosis in women with breast cancer treated with FAC chemotherapy. Observations similar to ours were reported in the study of Swart *et al*. where rs3732359 allele A *NR1/2* differentiated the patients into subgroups according to drug disproportion and therapy response^21^. This result was in concordance with Chew *et al*. observations that rs3732359 *NR1/2* was associated with a significant reduction in nadir hemoglobin, platelets and/or absolute neutrophil count (ANC) from baseline in cycle 1, either dependent or independent of the effects on the pharmacokinetics of docetaxel in nasopharyngeal cancer patients^48^. These results suggest the effect of rs3732359 on bone marrow hematopoietic capacity, and the ability to engage detoxification mechanisms in the presence of xenobiotics.

In our study there was a clear association of polymorphisms within *SLC22A16* gene engaged in doxorubicin transport with the risk of death and disease progression. Ota *et al*. and Faraji *et al*. shoved that polymorphisms within *SLC22A16* affected systemic pharmacodynamics of doxorubicin-based chemotherapy^49,50^. In our study, carriers rs7756222 CC and rs9487402 TG/GG *SLC22A16* have decreased OS. Furthermore, variant rs7756222 CC *SLC22A16* was the independent factor shortening PFS. Our results are consistent with the results of Lal *et al*. 2007, that SNPs in *SLC22A16* are associated with shorter OS and PFS in Asian breast cancer patients^51^. Furthermore, earlier studies confirmed the association of SNPs within *SLC22A16* with toxic side effects in chemotherapy in breast cancer patients^36,50,52^. Additionally, overexpression of SLC22A16 in cancer cells is associated with the increasing influx of doxorubicin into cell and correlates with increased sensitivity to cytotoxic effects of this drug^53^. In gastric cancer patients, SLC22A16 upregulation independently predicted poor OS and RFS, in early gastric cancer and poor OS in advanced gastric cancer^54^. Kunii *et al*. demonstrated also that SLC22A16 is a mediator of platinum uptake in cancer cells, and down-regulation of SLC22A16 is possibly one of the mechanisms of resistance against cisplatin in lung cancer^55^. The cited reports confirm that the genetic variants within *SLC22A16* gene influence the import efficacy of chemotherapeutic drugs into the cell, while toxicity generated by the changed drug level in the cell corroborated with the worst treatment response.

We present two independent genetic prognostic factors that significantly influenced the risk of shortened PFS (rs1824125 GG and CC rs11224560 of *PGR*) and RFS (rs3209896 AG *AKR1C3*) in breast cancer patients. To our knowledge, this is the first report of the potential interactions of both rs1824125 GG and rs11224560 CC progesterone receptor as well as rs3209896 AG *AKR1C3* (progesterone-related gene) with the survival and treatment response. Unfortunately, the functional data for *PGR*’s rs1824125 and rs11224560 are lacking, but the position of the studied variants in the gene regulatory sequences points out at their suspected role in expression control. It is commonly known, that the controlled expression of progesterone receptor is crucial for the breast tissue development, all the more that the *PGR* gene has two promoters and translational start sites and produces two isoforms, PR-A and PR-B. The PR-B is the positive regulator of the effects of progesterone, but PR-A antagonizes the effects of PR-B^56^. Balanced expression of both PR isoforms is required for maintaining mammary gland function, and any imbalance is associated with the increased risk of breast cancer. In this regard, it was shown that altered PR-A and PR-B balance distorts progesterone effects on breast cells, thus increasing breast cancer risk^57^.

In this study we presented the possible relationship between the risk of local recurrence and the rs3209896 AG variant in *AKR1C3*. AKR1C3 is phase I drug metabolizing enzyme implicated in drug resistance to chemotherapeutics including doxorubicin^58^. It plays a significant role in the deactivation of doxorubicin to doxorubicinol, a less active metabolite^59^. Polymorphisms in *AKR1C3* were studied as the risk factors for lung, prostate^60^, lymphoma^61^ and bladder cancer^62^. There are also reports regarding the role of *AKR1C3* variation in the risk of disease progression and mortality in B cell non-Hodgkin lymphoma^63^, as well as in the modulation of treatment toxicity and survival time in breast cancer patients^58^. However, the influence of rs3209896 polymorphism on cancer risk and chemotherapy response was considered only in two previous studies, but with no success. In the first, Asian breast cancer patients treated with doxorubicin-containing chemotherapy have shown no correlation between rs3209896 AG *AKR1C3* with chemotoxicity, PFS and OS^58^. In the second study, maternal and offspring genetic analyses of the *AKR1C3* gene did not reveal the association of rs3209896 *AKR1C3* in relation to childhood leukemia risk^61^. In our study the genetic-clinical linkage regarding the outcome in breast cancer patients treated with doxorubicin-containing FAC regime, on the other hand, did exist. Earlier study suggested that AKR1C3 belongs to the family of progesterone-related genes involved in the prereceptor metabolism of progesterone and believed it to be a weaker activator of PGR^64^. Given these data, our results further emphasize the clinical importance of progesterone signaling pathways. Study by Reding *et al*. showed that the variation in *AKR1C2* and *AKR1C3* genes could increase the risk of breast cancer among women who have used estrogen-progesterone therapy^65^. A putative role in breast cancer based on the AKR1C enzymes metabolizing progesterone into a 4-pregnene was described by Ji *et al*. These authors speculated that loss of AKR1C1 and AKR1C2, but not AKR1C3 in breast cancer, resulted in decreased progesterone catabolism which, in combination with increased PR expression, may augment progesterone signaling by its nuclear receptors^64^.

In our study the carriers of rs291593 CC *DPYD* had higher risk of disease recurrence. DPYD catabolizing 5-fluorouracil (5-FU), which is commonly used for the treatment of solid carcinomas^66–68^, and is also the component of FAC regime. A decrease in enzyme activity may lead to an increase in the half-life of 5-FU and an increased risk of dose-dependent toxicity^67–70^. The SNP rs291593 CC *DPYD* was described in the study of Kim *et al*., which focused on allele distribution in 150 Korean subjects^68^. Unfortunately, there are no literature data on the effect of rs291593 *DPYD* and correlation with the survival and treatment response.

Lastly, in our analyzed group of patients the polymorphism rs1054899 AG/AA *ALDH5A1* correlated with worse response to the FAC chemotherapy. ALDH5A1 is a component of cyclophosphamide metabolic pathway. A deficiency of this enzyme is a rare autosomal recessive neurologic disorder in which a serious gene mutation-related enzyme defect in the GABA degradation pathway causes a consecutive elevation of both GABA and gamma-hydroxybutyric acid (GHB)^71–75^. Apart from the neurotransmittory pathways, the ALDH5A1, alongside with ALDH1A1 and 3A1, participate in the transformation of cyclophosphamide, guiding the detoxification of aldophosphamide to inactive carboxyphosphamide^76^. It is widely accepted that inter-individual differences in the formation of cyclophosphamide metabolites may result from polymorphisms in genes that catalyze metabolic reactions, as well as from changes in their expression^77^. Although the data regarding the exact impact of the ALDH5A1 regulatory 3′UTR variation on the cyclophosphamide activity is lacking, there are reports suggesting the connection between enzyme expression and treatment prognosis. The group of Tian reported that low ALDH5A1 expression is an excellent predictive factor of poor prognosis in ovarian cancer (OC) and may play a crucial role in ovarian cancer progression. The positive association between expression of ALDH5A1 and prognosis was found in early and advanced stages of ovarian cancer patients. In grades II/III of ovarian cancer, a high mRNA level of ALDH5A1 was associated with improved OS^78^. Studies conducted on the Chinese epilepsy patients confirmed, that rs1054899 of *ALDH5A1* gene may play a role in the pharmacokinetics of valproic acid (VPA) with anticonvulsant properties^79^.

In our analyses, the shortened OS and PFS, and high risk of death and progression, were the results of advanced disease- tumor size over 20 mm in greatest dimension (i.e. T component greater than T1), infiltrated regional lymph nodes (i.e. N component different than N0) and the presence of distant metastases (M1). These observations are in accordance with the traditional perception of the stepwise progression of breast cancers, from small, not yet spread tumors, to larger more aggressive ones^80^. The tumor size (T component) is seen as one of the most crucial factors of response to treatment. In the study of Goorts *et al*.^81^ the clinical tumor size (cT) was the strongest predictor of achieving the pathological complete response (pCR), seen as the absence of residual cancer, in the group of breast cancer patients after neoadjuvant chemotherapy. In this study, higher cT-stages had strong, significant lower pCR rates, independently of grade and progesterone, estrogen receptors and HER2 status. Also, the newest, eight edition of Cancer Staging Manual^82^ confirms that the whole TNM staging system for breast cancer is the estimate of total tumor volume, described by the maximum dimension of the tumor mass, without inclusion of additional small satellite foci surrounding the main tumor^83^.

The presence of regional lymph node metastases is another acknowledged factor of worse prognosis in breast cancer patients. According to the newest guidelines, the classification of nodes is made upon the size of the largest tumor deposit, and the sum of infiltrated lymph nodes results in the final N-value^82^. Generally, the scale of nodes involvement is reflected in worsened patients’ prognosis, seen - among the others - in reduced survival time^84^. Similarly, the presence, location and extent of distant metastases are unequivocally responsible for poor prognosis, with estimated 90% breast cancer-related deaths being due to metastatic dissemination^85^. Such drastic correlation was seen also in our analyses, where the presence of distant metastases was the strongest, also statistically, survival-reducing factor. This situation seems to reflect the discordances between the primary and secondary tumors, regarding the clinical and biological features that change the treatment response rate between those sites. Also, there are complicated patterns linking the metastatic spread with the location of primary mass, the patients’ age and also with the unforeseen impact of surgical procedures on main tumor mass, which may enhance the growth of dormant micrometastases in different organs^85^.

In our study the breast cancer negative status of progesterone receptor was the determinant of high risk of recurrence, shorter TFFS and lack of response to treatment. The status of PR, estrogen receptors (ERs) are among the biological factors, aside from HER2 expression and grade, that had been incorporated into the breast cancer staging system. The goal of such approach was to combine the latest biological knowledge with up-to-date clinical research in hope to establish the simple but accurate staging guideline that defines the prognosis with the most possible precision^86^. The lack of progesterone receptor expression in breast cancer has been in recent years repeatedly reported as the factor for poor treatment outcome, which is consistent with our results. In the work of Purdie and others, the group of PR-negative patients, even the subgroups that otherwise would have a good prognosis- i.e. ER-positive and without lymph node infiltration, had significantly shortened the 5-year breast cancer specific survival, with or without application of chemotherapy^87^. In the cited work, the PR expression was the independent prognostic factor, more powerful than ER status. Similar results came from the study of van Mackelenbergh *et al*., where the ER-positive/PR-negative breast cancers showed better initial response to treatment, even though eventually the long term survival after neoadjuvant chemotherapy had been significantly reduced^88^. The complexity of impact of hormone receptor statuses on treatment outcome could arise from the cross-talk mechanisms existing between estrogen and progesterone receptors. When the PR is absent, the estrogen receptor recruits specific cofactors and binds to estrogen response elements in chromatin. The result is the activation of pathways that leads to cell proliferation^89^. Such crosstalk was illustrated in the work of Mohammed *et al*.^90^, who pointed out, that in breast cancer PR modulates ER behavior, and its expression can be seen as a marker of ER function.

The analyses of genetic and clinical factors could be useful in the attempt to establish the complex combined genetic-clinical models for the patients’ preselection into more uniform groups with similar prognosis with regard to death, disease progression, recurrence, development of metachronic breast cancer and overall treatment response. In order to do that, we performed cumulative analyses, and selected the groups of patients with the seemingly worst prognosis, who carried the highest number of unfavorable factors in a given setting. For each of the analyses the clinical component pointed out the advancement of the disease. The picture was further completed by the addition of genetic modifiers. Because of the frequent lack of data regarding the exact impact of studied genetic variant on gene or protein function, every interpretation of cumulative model must be done with great caution. Nonetheless, the OS and the highest risk of death was the obvious result of advanced TNM stages, but the presence of two variants in doxorubicin transporter *SLC22A16* in this model emphasize the importance of optimal drug inflow to the cells. Furthermore, the activity of the main detoxification switch NR1/2 in OS model seems to be the player in mediating death risk. We could assume, that the worse treatment response in this regard may be the reflection of overactive detoxification routes of any of FAC drugs, which decrease the drugs’ therapeutic concentration. It should be noted, that the strength of this model is further enhanced by the lack of death incidences in the group of non-carriers of high-risk factors.

The group with the worst prognosis regarding cancer progression was also characterized by the disease advancement (metastases) and polymorphic variant in doxorubicin importer gene *SLC22A16*. However, in this model the picture was completed by the presence of two SNPs in progesterone receptor gene. The ‘double hit’ as such, similarly as for the *SLC22A16* in OS analysis, point out the relations between physiological, metabolic and signaling pathways of progesterone and the metastatic ability of breast cancer cells, as well as the revival of dormant micrometastases. The progesterone signaling routes, seen as the negative PR receptor status in cancer cells, emerged also as the component of the worst-case scenario in recurrence-free survival analysis. In this situation however, high risk of recurrence was concomitantly dependent on the modification in 5-fluorouracil catabolizer *DPYD*, as well as in *AKR1C3*, the component of both doxorubicin and progesterone metabolic mechanisms. This result once again emphasizes the importance of progesterone signaling and the activity of detoxification machinery, that ensures the optimal therapeutic drugs’ concentration.

To our knowledge, this is the first study to evaluate the prognostic value of 3′UTR polymorphisms of ADME genes in the context of overall treatment response in breast cancer. However, the clinical determinants as high risk factors dominated the genetic ones in this analysis. Unsurprisingly, the results showed that PR tumor negative status, together with the lack of surgical procedures, which in turn is the direct consequence of patient’s poor condition, are the predictors of negative events such as death, progression, recurrence and the development of another breast cancer. The only genetic component in this model, variant in *ALDH5A1*, point out the cyclophosphamide catabolic mechanisms. Additionally, the strength of this model is backed by the results of TFFS analysis. While the original treatment response in this work was estimated within 10 months since the beginning of chemotherapy, the obtained separation of patients into groups in regard to the number of unfavorable factors enabled predicting long-term survival. Following this analysis we report the unprecedented, over 14-fold reduction in median survival between the groups with the best and worst prognosis.

In conclusion, the normal germline variants commonly present in the population are important factors determining the response to treatment. The study shows that polymorphisms are an independent prognostic predictor factor of survival in breast cancer woman. Furthermore, we observed the effect of the accumulation of multiple unfavorable genetic and clinical factors on poor survival prognosis and overall treatment response. The results suggest that germline polymorphisms influence the pharmacokinetics of doxorubicin. Our study indicates the strongest associations between overall survival, progression-free survival and genetic polymorphisms in *SLC22A16* gene engaged in doxorubicin transport. In addition, SNP in the *AKR1C3*, a gene involved in the doxorubicin metabolism pathway, has an effect on recurrence-free survival. To sum up, the presence of adverse genetic and clinical factors increases the risk of poor outcome of treatment response in Polish women with breast cancer who were treated with FAC regime. This study implicates that selection of patients based on the cumulative unfavorable factors models may be helpful in predicting prognosis in regard to death, progression or recurrency.

## Data Availability

The data and material are available after the approval by Maria Sklodowska-Curie National Research Institute of Oncology, Gliwice, Poland.
